# The novel p.A30G *SNCA* pathogenic variant in Greek patients with familial and sporadic Parkinson's disease

**DOI:** 10.1111/ene.16562

**Published:** 2025-01-29

**Authors:** Ioanna Alefanti, Christos Koros, Viktoria Tsami, Athina Maria Simitsi, Chrisoula Kartanou, Nikolaos Papagiannakis, Maria Bozi, Roubina Antonelou, Matina Maniati, Ann‐Kathrin Hauser, Stefanos Varvaressos, Anastasios Bonakis, Konstantinos Lourentzos, Periklis Makrythanasis, Sokratis G. Papageorgiou, Christos Proukakis, Constantinos Potagas, Thomas Gasser, Georgios Koutsis, Georgia Karadima, Leonidas Stefanis

**Affiliations:** ^1^ 1st Department of Neurology Eginition Hospital, National and Kapodistrian University of Athens Athens Greece; ^2^ Neurogenetics Unit, 1st Department of Neurology Eginition Hospital, Medical School, National and Kapodistrian University of Athens Athens Greece; ^3^ Laboratory of Neurodegenerative Diseases Biomedical Research Foundation of the Academy of Athens Athens Greece; ^4^ 2nd Department of Neurology Attikon Hospital, National and Kapodistrian University of Athens Athens Greece; ^5^ Department for Neurodegenerative Diseases Hertie Institute for Clinical Brain Research, University of Tübingen and German Center of Neurodegenerative Diseases (DZNE) Tübingen Germany; ^6^ Department of Medical Genetics National and Kapodistrian University of Athens Athens Greece; ^7^ Department of Clinical and Movement Neurosciences UCL Queen Square Institute of Neurology London UK

**Keywords:** Greek population, p.A30G, Parkinson's disease, *SNCA*

## Abstract

**Background:**

The p.A53T variant in the *SNCA* gene was considered, until recently, to be the only *SNCA* variant causing familial Parkinson's disease (PD) in the Greek population. We identified a novel heterozygous p.A30G (c.89 C>G) *SNCA* pathogenic variant in five affected individuals of three Greek families, leading to autosomal dominant PD. This study aims to further explore the presence and phenotypic expression of this variant in the Greek PD population.

**Methods:**

Restriction fragment length polymorphism (RFLPs) was used for genotyping of 664 Greek PD cases. Detailed clinical information was obtained for the carriers and p.A30G‐positive samples underwent haplotype analysis.

**Results:**

We identified 10 additional p.A30G‐positive PD patients (1.5%), of whom 4 were sporadic cases (0.9%). They manifested typical Parkinsonian motor dysfunction, with a mean age of onset of 51.7 years (range: 33–62) and a broad spectrum of non‐motor symptoms. The absence of affected first degree relatives in four out of ten index cases, and the presence of a phenocopy in an additional family, suggest that the p.A30G variant manifests reduced penetrance. The common haplotype among the p.A30G carriers confirmed a founder effect. Furthermore, two asymptomatic carriers were identified, with possible premotor manifestations.

**Conclusions:**

These findings underscore that the p.A30G *SNCA* pathogenic variant represents an important, albeit rare, cause of genetic PD in the Greek population. This is the first time in which a genetic synucleinopathy, with a variant in the *SNCA* gene, is clearly linked to an appreciable frequency of sporadic PD in a particular population.

## INTRODUCTION

Parkinson's disease (PD) is a complex syndrome, clinically characterized by bradykinesia, resting tremor, muscle rigidity and, in advanced stages, postural instability, as well as several non‐motor manifestations [1]. The etiology of the disease is heterogeneous and is thought to involve a combination of genetic and environmental components. Approximately 10% of the patients exhibit familial aggregation, suggesting a genetic cause [2]. The genetic determinants of PD range widely, from highly penetrant rare DNA variants leading to monogenic or Mendelian forms of disease, to variants with intermediate or low penetrance, consistent with genetic risk factors [3, 4]. The *SNCA* gene encoding for the presynaptic protein α‐synuclein is the first gene identified with variants linked to familial PD, in cases of Italian or Greek origin [5]. Since the first identification of the p.A53T pathogenic variant, several other missense variants and multiplications (duplications and triplication) of the *SNCA* gene have been identified, enhancing the understanding of the disease's pathogenicity [6, 7]. In the Greek population, until recently, the p.A53T pathogenic variant was considered to be the only *SNCA* variant causing familial PD^8^.

We recently reported [9] a novel heterozygous p.A30G (c.89 C>G) *SNCA* variant in five affected individuals of three unrelated Greek families, causing autosomal dominant PD. The affected individuals exhibited typical PD phenotypes, including levodopa‐responsive Parkinsonism, age of onset varying from 36 to 80 years old (the latter in a paucisymptomatic individual) and various prominent non‐motor symptoms, such as cognitive decline, psychiatric manifestations, orthostatic hypotension and REM Sleep Behavior Disorder (RBD). All carriers harbored the same haplotype suggesting a “founder effect.” Furthermore, biophysical analyses demonstrated potential pathogenic properties of the p.A30G variant protein. These findings enhanced prior knowledge regarding aspects of pathogenicity of α‐synuclein, while they also identified another *SNCA* pathogenic variant in the Greek population, apart from p.A53T, that causes autosomal‐dominant PD.

The purpose of this study is to provide further evidence of the presence of this new pathogenic variant in the Greek PD population, and to expand the understanding of its phenotypic spectrum. The genetic and clinical findings of 12 new variant carriers are reported, of which 2 are asymptomatic cases.

## MATERIALS AND METHODS

### Patients

We screened for the p.A30G *SNCA* variant in an additional Greek cohort of 664 unrelated PD patients (393 males and 271 females), 214 of which had a positive family history with at least one first‐ or second‐degree relative with PD and 447 sporadic PD cases. Among the sporadic cases, 178 presented with an early age of disease onset (<50 years old) and 269 with mid‐late age of onset. The mean age at onset was 55.4 ± 12.1 years and the mean age of first examination was 61.2 ± 11.5 years. Table 1 demonstrates the demographic and clinical information of the cohort.

**TABLE 1:** Demographic and clinical data of Greek PD patients.

	Total cohort	Familial cases	Familial cases with mid‐late AAO	Familial cases with early AAO	Sporadic cases	Sporadic cases with mid‐late AAO	Sporadic cases with early AAO	Inheritance information NA
*N* (%)	664 (100%)	214 (32.2%)	144 (21.7%)	70 (10.5%)	447 (67.3%)	269 (40.5%)	178 (26.8%)	3 (0.5%)
p.A30G positive patients (%)	10 (1.5%)	6 (2.8%)	3 (2.1%)	3 (4.3%)	4 (0.9%)	2 (0.7%)	2 (1.1%)	—
Sex (M/F)	393/271	120/94			271/176			
Mean age (years) at onset ± SD (Range)	55.4 ± 12.1 (18–85)	56.5 ± 12.4 (23–85)			54.9 ± 12 (18–80)			
Mean age (years) at examination ± SD (Range)	61.2 ± 11. 5 (19–86)	62.1 ± 11.5 (35–86)			60.9 ± 11.5 (19–86)			

Abbreviations: AAO, Age at onset; NA, Not available; SD, Standard Deviation.

The patients were recruited from the Neurogenetics Unit's outpatient clinic of the First Department of Neurology of the National and Kapodistrian University of Athens and the specialist movement disorders outpatient clinics of the First and Second Department of Neurology of NKUA. PD was diagnosed based on the Movement Disorder Society (MDS) clinical diagnostic criteria [10]. The study was approved by the Eginition and Attiko Hospital ethics committees. All participants provided informed consent before participating in the performance of the molecular genetic testing.

A clinical evaluation of variant carriers was performed using the Movement Disorders Society (MDS) Unified Parkinson's Disease Rating Scale (MDS‐UPDRS). Cognitive function was evaluated by the Mini‐Mental Status Examination (MMSE) [11] or the Montreal Cognitive Assessment (MoCA) [12]. Olfactory dysfunction was assessed using the identification part of the 12‐item Sniffin' Sticks (cutoff: 9; cutoff: 7 for patients >55 years) [13]. RBD was assessed by the REM sleep behavior disorder Questionnaire [14]. The Epworth Sleepiness Scale (ESS) was used to assess daytime sleepiness [15], and the Scales for Outcomes in Parkinson's Disease‐Autonomic questionnaire (SCOPA‐AUT) for autonomic dysfunction [16]. The geriatric Depression Scale (GDS) was used for the evaluation of depression [17]. In some cases, the above evaluation was not complete, due to unavailability of the subjects.

### Genetic studies

**FIGURE 1:**
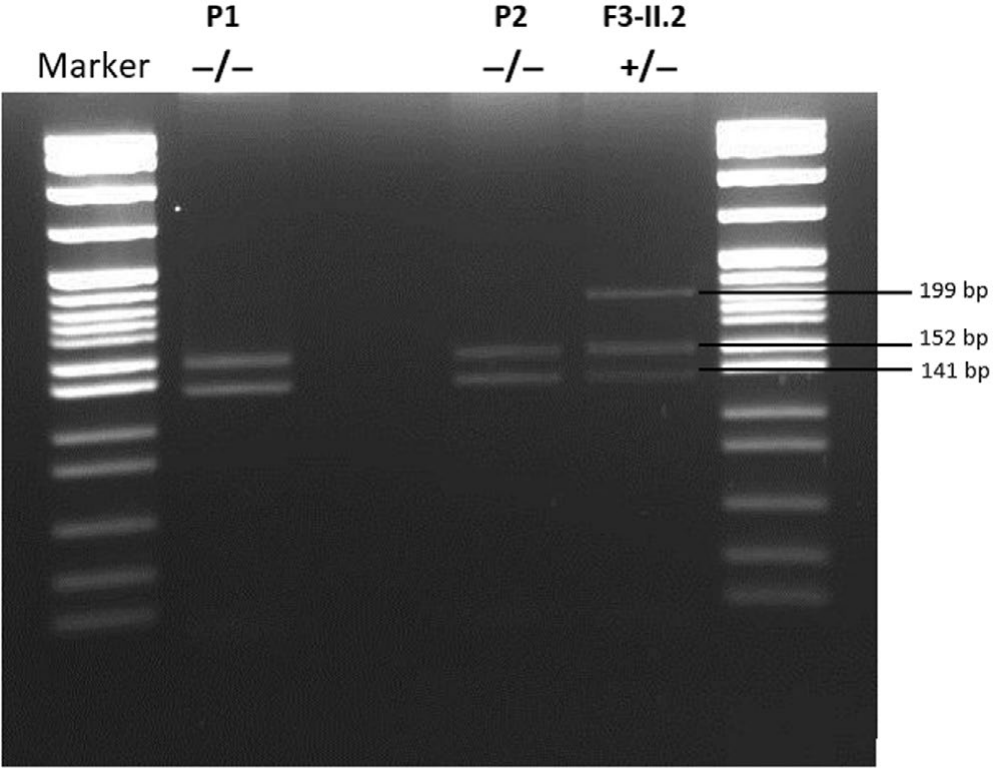
Restriction digestion. The digested products of one heterozygous p.A30G variant carrier (+/−) (index case F3‐II.2) and two cases (Patient 1 [P1], Patient 2 [P2]) who tested negative for the p.A30G variant (−/−). The marker used was pBR322 DNA‐Mspl digest.

Genomic DNA was isolated from peripheral blood leukocytes by standard procedures. We screened for the p.A30G *SNCA* variant by performing Restriction Fragment Length Polymorphism (RFLP) with BbvI (R0173S) restriction enzyme (Neurogenetics Unit, First Department of Neurology and Laboratory of Neurodegenerative Diseases, Biomedical Research Foundation of the Academy of Athens) according to the manufacturer's protocol (New England Biolabs) as previously described [9] (Figure 1). Haplotype analysis (Department for Neurodegenerative Diseases, Hertie Institute for Clinical Brain Research, University of Tübingen and German Center of Neurodegenerative Diseases) of the available family members was conducted by utilizing short tandem repeat markers across the *SNCA* locus (D4S1553, D4S400, D4S2932, rs3068933, rs10586902, NACP‐REP1, rs59666810, rs57454775, rs138994786, and D4S1538).

## RESULTS

**FIGURE 2:**
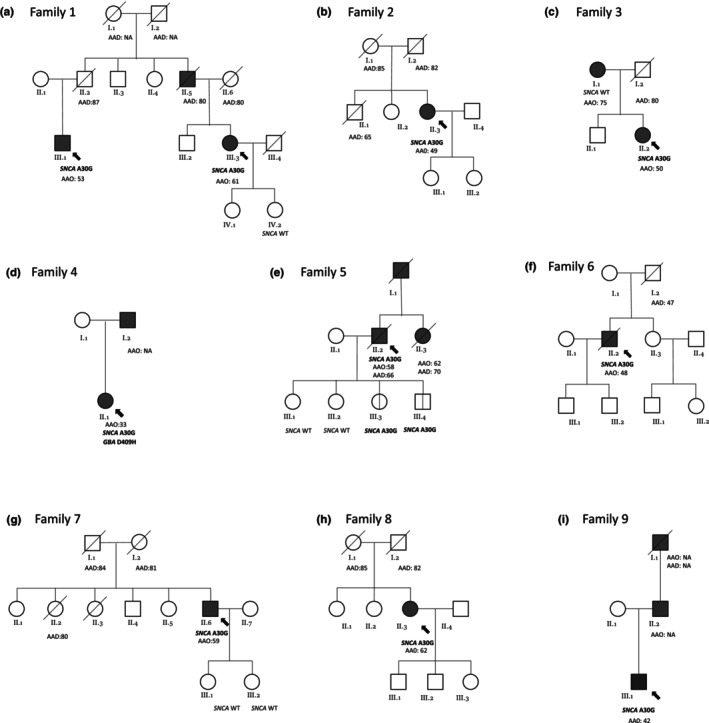
Pedigrees of the seven p.A30G families. (a) Pedigree of Family 1, (b) Pedigree of Family 2, (c) Pedigree of Family 3, (d) Pedigree of Family 4, (e) Pedigree of Family 5, (f) Pedigree of Family 6, (g) Pedigree of Family 7, (h) Pedigree of Family 8, (i) Pedigree of Family 9. Circles denote females and squares denote males. Filled black symbols indicate affected individuals with Parkinson's disease (PD), white symbols unaffected individuals. The vertical lines inside the symbols represent clinically unaffected carriers of the variant that could later exhibit symptoms. The arrows indicate the index case of each family. Crossed‐out individuals are deceased. AAD, age at death; AAO, age at onset; NA, not available; WT, wild type.

**FIGURE 3:**
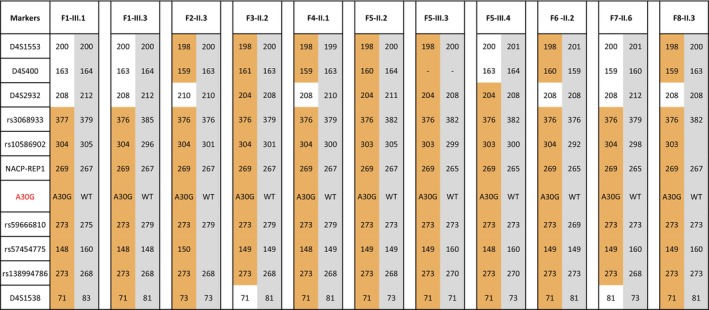
Haplotype analysis. A common haplotype across the SNCA locus revealed among the 11 tested p.A30G SNCA carriers, marked in yellow.

In total, of the 664 patients screened, 10 p.A30G‐positive carriers of the variant were identified (10/664, 1.5%). Of note, 4/447 sporadic cases (0.9%) harbored the pathogenic variant. Table 2 summarizes the demographic and clinical findings of the 10 p.A30G patients. The pedigree charts of the p.A30G‐positive families are demonstrated in Figure 2. The mean age at onset of the disease was 51.7 ± 9.2 years (median: 52.5 years) with a range of 33–62 years and the mean disease duration was 7.2 ± 4 years. Five of the patients were females and five were males. Variant carriers underwent haplotype analysis at the *SNCA* locus that revealed a shared haplotype (Figure 3). The shared haplotype is between rs3068933 (4:89486111) and rs138994786 (4:90107743), including approximately 621.630 bp.

**TABLE 2:** Demographic and clinical features of p.A30G patients of the seven Greek families.

Patients	Family 1 (III.3)	Family 1 (III.1)	Family 2 (II.3)	Family 3 (II.2)	Family 4 (II.1)	Family 5 (II.2)	Family 6 (II.2)	Family 7 (II.6)	Family 8 (II.3)	Family 9 (III.1)
Sex	Female	Male	Female	Female	Female	Male	Male	Male	Female	Male
Age at onset (years)	61	53	49	50	33	58	48	59	62	42
Age at examination (years)	63	61	62	56	46	64	59	65	67	43
Duration (years)	3	8	13	6	13	6	11	6	5	1
Initial symptom	Bradykinesia	Rigidity, bradykinesia	Rest tremor	Rigidity, bradykinesia	ΝΑ	Rigidity	Rigidity, bradykinesia	Rest tremor	Rest tremor	Rigidity, bradykinesia
Asymmetry of initial symptom	−	+	+	+	+	+	+	+	+	+
Bradykinesia	+	+	+	+	+	+	+	+	+	+
Rigidity	−	+	+	+	+	+	+	+	+	+
Tremor	−	−	+	−	+	+	−	+	+	+
Postural instability	−	−	−	+	+	+	+	−	−	−
Response to levodopa	+	+	+	+	+	+	+	+	+	+
Complication with treatment	+	+	+	+	+	+	+	+	+	−
UPDRS III score	NA	30 (off)	24 (on)	9 (on)	NA	40 (on)	62 (off)	16 (on)	32 (on)	NA
Autonomic dysfunction	Constipation	Constipation	Constipation, urinary incontinence	Urinary incontinence, constipation	Constipation	−	Urinary incontinence	Constipation, dysphagia	Constipation	Mild constipation
Olfactory deficits	+	+	+	+	NA	−	+	+	+	+
Sleep disturbances	RBD symptoms	Daytime Sleepiness, RBD symptoms	Daytime Sleepiness, RBD symptoms	NA	Sleep disturbances	RBD	Daytime Sleepiness	Possible RBD	RBD	−
Cognition	MMSE: 27/30	MoCA: 22/30	MoCA: 10/30	MMSE: 30/30, MoCA: 29/30	Dementia	MMSE: 20/30	MoCA:16/30	MoCA: 23/30	MoCA: 14/30	MMSE: 30/30
Mood disorders	Depression	Depression	Depression, anxiety, apathy	Depression, anxiety	Depression	−	Depression, apathy	Anxiety	Depression	Anxiety
Psychotic symptoms	−	Hallucinations	−	−	NA	−	Hallucinations/delusion	−	Hallucinations	−
Other features	−	Pain sensation	Pain sensation	Dystonia	Dystonia, seborrheic dermatitis of the scalp	NA	Pain sensation, fatigue	−	−	Dystonia

Abbreviations: MMSE, Mini‐Mental Status Examination; MoCA, Montreal Cognitive Assessment; NA, not available; RBD, Rapid eye movements sleep behavior disorder; UPDRS, Unified Parkinson's disease Rating Scale.

All patients presented with typical Parkinsonian symptoms. The majority of the patients presented with bradykinesia and rigidity, while resting tremor was absent in four symptomatic p.A30G carriers (Table 2) even years later in the disease course. Notably, dystonia coexisted with Parkinsonism in three of the cases (index case F3‐II.2, F4‐II.1, and F9‐III.1). Furthermore, all patients had good initial response to levodopa treatment but most developed early motor complications and four of them (F1‐III.1, F3‐II.2, F4‐II.1, and F6‐II.2) eventually underwent duodopa pump therapy. Significant cognitive deficits were present in 5/10; three (F5‐II.2, F6‐II.2, and F8‐II.3) had moderate and two (F2‐II.3 and F4‐II.1) had severe cognitive impairment. Index case F8‐II.3 manifested cognitive deterioration 4–5 years after disease initiation, while for the rest of the cases, this occurred after 6–10 years. Autonomic dysfunction, psychiatric symptoms, olfactory deficits, and sleep disturbances (RBD and daytime sleepiness) were reported in the majority of the patients (Table 2). Hallucinations were mentioned by three of the patients (F1‐III.1, F6‐II.2, and F8‐II.3). It is noteworthy that families 2, 6, 7, and 8 (Figure 2) reported no PD‐related family history, and the father of patient F1‐III.3 only developed PD in his 80s, while the parents of the affected cousin (III.1) of the index case never manifested the disease. Additionally, the mother of index case F3‐II.2 tested negative for the p.A30G variant, even though she manifested PD in her 70s, while the patient's father had not manifested signs of Parkinsonism by the time he died in his 80s. Index case F4‐II.1 was also a known carrier of the D409H *GBA* variant, identified through targeted RFLP assessment for *GBA* variants common in the Greek population, as previously described [8]. The detailed clinical features of the 9 p.A30G families are provided in the supplemental material (Data S1).

### Asymptomatic carriers

Members of families 1, 5, and 7 were recruited and underwent presymptomatic genetic testing for the p.A30G variant. The variant was confirmed in two (F5‐III.3 and F5‐III.4) out of four children of index patient 1. Case F1‐IV.2 and cases F7‐III.1 and F7‐III.2 tested negative for the variant.

The two variant carriers underwent neurological examination. The first one was a woman in her 40s (F5‐III.3) with an 8‐year education and the second was a man in his 40s (F5‐III.4) with a 12‐year education. Upon examination, no motor symptoms or signs were revealed. Regarding non‐motor features, subject F5‐III.3 manifested psychiatric symptoms, such as mild depression (GDS questionnaire: 5) and anxiety. She mentioned sleep disturbances and scored an 8 on the RBD questionnaire and a 7 on the ESS. Examination with polysomnography (PGS) revealed periodic leg movements, but no RBD. Olfactory testing was normal (Sniffin' Sticks test: 10/12) and the MoCA score was 25/30. Subject F5‐III.4 had olfactory deficits (Sniffin' Sticks test: 6/12) and sleep disturbances with possible REM sleep behavior disorder (RBD questionnaire: 7) and an ESS score of 7. The MoCA score was 26/30.

## DISCUSSION

In the present study, the genetic and clinical features of 12 carriers of the novel heterozygous p.A30G *SNCA* variant from 9 unrelated Greek families are described. The results are in accordance with the original study [9] and contribute deeper insights into the phenotypic spectrum of p.A30G *SNCA*‐associated PD.

The 10 newly identified PD patients carrying the p.A30G variant manifested a typical PD phenotype including motor and non‐motor symptoms. The mean age of onset was 51.7 ± 9.2 (mean ± SD) years with a range of 33–62 years of age. The origin of the nine affected families was from geographically distinct areas in Greece, with no suggestion of common regional ancestry. This contrasts with the p.A53T variant, which is invariably present in subjects of Peloponnesian origin. The p.A30G variant carriers shared the same haplotype identified in the original study [9], indicating that the variant originated from a common ancestor in the Greek population. Motor symptoms are similar to those of typical idiopathic PD, with rest tremor being less common. All patients developed a variety of non‐motor manifestations including autonomic dysfunction, olfactory deficits, sleep disturbances (RBD and daytime sleepiness), as well as mood disorder (depression and anxiety). Cognitive problems were present in about half of the cases. Importantly, index case F4‐II.1, identified to harbor both *GBA1* and *SNCA* variants, exhibited an earlier age of onset and a very severe disease course compared to the rest of the cases. Interestingly, one other unpublished case from our p.A53T cohort was also found to harbor an additional PD‐related variant in *GBA1*, had an age of onset below 30, and a similar, very aggressive course, leading to death prior to the age of 40. Prior research has shown that the presence of a *GBA1* variant predisposes to an earlier disease onset and a more severe disease course [18, 19]. However, the exact effects of *GBA1* mutations when co‐occurring with *SNCA* or *LRRK2* mutations remain to be elucidated [20, 21, 22].

It is interesting to note that in the current study, four of the cases reported no PD‐related family history. In addition to that, parents of some probands are reported to manifest disease at late ages, beyond 70, suggesting that this genetic disease may manifest in a quite delayed fashion in certain cases; one has to be wary of course of phenocopies, as occurred in the case of Family 3, and it has to be stressed that these late‐onset cases have not been genetically confirmed. Some evidence pointing to reduced penetrance of the p.A30G *SNCA* pathogenic variant had already been highlighted in the original study [9]. Considering that the majority of cases presented here do not have a clear autosomal dominant pattern of inheritance, it would appear that the p.A30G *SNCA* variant shows modest penetrance. Both *SNCA* missense variants and copy number variants have been previously described to show incomplete penetrance [23, 24, 25]. Given the reduced penetrance of the p.A30G variant, it is likely that it would also appear in unaffected control individuals, even at advanced ages, as in apparently obligate carriers, asymptomatic parents of affected carriers in the present study. In our original work, none of the 377 examined Greek controls harbored the variant [9], but it has to be taken into account that such controls are carefully selected to not have PD family history in the first or second degree. In an updated search we performed, the p.A30G variant represents only 2 out of 1.180.014 European (non‐Finnish) alleles, according to the gnomAD v4.1.0 [26], although no more precise ethnicity details are available. Such data, including the common haplotype found in the original study and confirmed here in the variant carriers, suggest that the variant has arisen due to a founder effect and may be largely restricted to the Greek population.

To elucidate the functional properties of the p.A30G variant, in our original study, we conducted biophysical studies of the recombinant protein. We found that the p.A30G substitution causes a “double‐hit,” which might explain its pathogenicity. The p.A30G variant's α‐helical structure is disrupted, slightly perturbing its ability to bind to membranes; furthermore, amyloid fibril formation is slightly enhanced [9]. The decreased α‐helical structure and impaired membrane binding were confirmed in another report [27]. Similarly, the p.G51D and, especially, the p.A30P, variants have significantly disrupted membrane binding ability [28]. Notably, p.A30P α‐synuclein fibrillizes slower than the wild type (WT), but its oligomerization is accelerated [28, 29, 30]. The H50Q, A53E, and A53T substitutions have a very mild effect on the ability of α‐synuclein to bind to lipid membranes, whereas, p.A53T, p.G51D, and p.H50Q promote α‐synuclein fibrillization [28]. The p.E46K variant exhibits a distinct profile regarding membrane binding, demonstrating enhanced affinity compared to WT, and forms filaments at a significantly faster rate, comparable to that of p.A53T [31]. Additionally, biochemical studies of p.V15A α‐synuclein (another variant localizing on the hydrophobic side of the first amphipathic helix of the N‐terminal region, similar to p.A30P and p.A30G) showed a modest decrease in phospholipid binding that was intermediate between that of p.A30P and WT. [32, 33] Furthermore, this variant aggregates more than the WT in seeding assays [33], when incubated with liposomes [32], as well as in cultured iPSC‐derived dopaminergic neurons [34]. Thus, the two‐hit model we have proposed for p.A30G may also be applicable to V15A. Finally, the newly identified p.T72M variant, positioned in the non‐amyloid‐β component (NAC) part of α‐synuclein, which is crucial for fibril assembly, was found to aggregate earlier and more robustly than the WT protein [35].

The phenotypic spectrum of p.A53T‐associated PD has been well described in the literature. Compared to p.A30G, the mean age at onset of p.A53T‐positive patients is 6 to 13 years earlier (average of 45 years old), with patients exhibiting a more aggressive disease course [36]. The motor features of both *SNCA* variants are similar to those of typical PD with the exception of resting tremor, which was reported as less common among p.A53T [37] and p.A30G carriers [9]. Patients with both variants show a good initial response to levodopa treatment, followed by motor fluctuations. Additionally, the p.A53T variant is responsible for a variety of non‐motor symptoms, with the most prominent being olfactory and autonomic dysfunction, as well as dementia [25, 38]. Similar non‐motor symptoms are present in p.A30G‐associated PD. The p.A30P is another missense *SNCA* variant identified only in one German family [39] with a substitution at the same amino acid position as the p.A30G. The phenotype of patients carrying p.A30P reveals similarities with that of p.A30G with regards to motor symptoms and possibly cognitive decline, although the cases are very few [40]. Larger cohorts are needed in order to gain insights into the clinical phenotype of these two *SNCA* variants at the A30 site.

Other point mutations, although rare, have been identified in several cases, often associated with early‐onset PD and unique clinical profiles. A relatively novel p.V15A variant has been identified in one Turkish, one Italian, and two Japanese families, characterized by relatively early age of onset, around 40s to 50s, and a Parkinsonian phenotype accompanied by cognitive decline and visual hallucinations [33, 34, 41]. Another variant, p.E46K, identified in Spanish families, causes a PD phenotype with variable age of onset and features of Lewy body dementia [42, 43]. The p.H50Q variant has been linked to sporadic and familial PD, but subsequently, its pathogenicity has been questioned [44, 45, 46]. Carriers of the p.G51D variant exhibit a phenotype of atypical PD with multiple system atrophy‐like features (MSA) [47, 48, 49]. The p.A53E is a rare cause of early onset PD in the Finish population and has been identified only in one Canadian family [50, 51, 52]. p.A53V is a pathogenic variant causing Parkinsonism and cognitive decline in both the homozygous and heterozygous states in Asian populations [53, 54]. Recently, a p.T72M variant was described in two Turkish families co‐segregating with late‐onset autosomal dominant PD^35^. Both duplications and triplications in the *SNCA* gene have been identified in familial PD [55, 56, 57, 58]. Disease severity is directly correlated with the *SNCA* gene dosage. *SNCA* triplications which were first identified in the Iowa kindred are characterized by early‐onset, highly penetrant disease with severe non motor manifestations including dementia [57, 58, 59, 60]. In contrast, SNCA duplication carriers have a milder PD phenotype with later onset of symptoms and slower disease progression, and may manifest as sporadic cases due to decreased penetrance, similar to the p.A30G variant [55, 56, 61, 62, 63].

The present study also describes the clinical findings of two asymptomatic siblings, carriers of the p.A30G variant. The two asymptomatic carriers were in their 40s and were younger than the earliest reported age of onset in their families. On clinical examination, one of them demonstrated olfactory deficits and sleep disturbances with possible RBD. These clinical symptoms are possibly indicative of the premotor phase of PD and could be attributed to an early manifest neurodegenerative process, as previously described [64]. However, such an assumption remains hypothetical given the lack of further objective evidence of dopaminergic loss (through DaTSCAN) or of ongoing aberrant α‐synuclein aggregation (through the Cerebrospinal Fluid α‐synuclein Seeding Amplification Assay (CSF AS SAA)). Similar clinical phenotypes have also been described in asymptomatic carriers of other *SNCA* missense variants. Regarding p.A53T, olfactory dysfunction and possible REM sleep behavior disorder have been reported to precede motor symptoms [25, 65]. However, RBD was not proven to occur in asymptomatic p.A53T carriers when they underwent formal PSG (polysomnography), underscoring the need for PSG confirmation of RBD diagnosis [66].

Based on the present findings, the p.A30G variant, although uncommon, represents an important cause of PD (10/664 cases tested, 1.5%) in a Greek population which, although favoring familial and early onset cases, does have a broad demographic representation. Importantly, the variant is identified not only in familial but also in sporadic PD (4/447 cases tested, 0.9%), even in mid‐late onset sporadic cases (2/269 cases tested, 0.7%) (Table 1). In a highly select Greek population of either familial PD or early onset PD (age of onset at or below age 50), we estimated the frequency of p.A53T SNCA at 4.5%, while the frequency increased to 15.4% among those with both dominant family history and early age at onset. In the latter more restrictive setting, descent from the Peloponnese raises the suspicion of the disease to almost 100% [8]. In another Greek cohort including both sporadic and familial cases with PD or PD plus dementia, the p.A53T variant was relatively common (1.8%) and was always associated with familial disease [67]. This has led to the recommendation that in the Greek population all familial cases should be tested for the p.A53T *SNCA* variant, especially if there is an early age of onset and a clear autosomal dominant pattern of inheritance and the subject is of Peloponnesian descent. In the case of p.A30G, current data indicate that basically all PD cases of Greek origin should be tested for the variant, given that the age of onset is not that low and that sporadic cases are clearly present in this population, while there is also no specific geographical distribution. These findings are especially noteworthy, as it is the first time that a genetic synucleinopathy, with a pathogenic variant in the *SNCA* gene, is found to be responsible for an appreciable frequency of sporadic PD in a particular population.

Identifying with such wider screening, as performed here, additional carriers of the p.A30G *SNCA* variant will be very important to further characterize the phenotype of symptomatic and asymptomatic carriers and determine the precise progression of *SNCA*‐related neurodegeneration in the preclinical and clinical disease stages of PD. With the advent of potential neuroprotective therapies targeting α‐synuclein, if such populations of genetic synucleinopathies were enlarged, they could be used for small proof of principle clinical trials, with the obvious advantage being that they represent subjects in whom pathogenic α‐synuclein is certainly etiologically linked to the disease.

To conclude, studies like the present are crucial in implementing a more individualized genetic testing protocol, based on the ethnic origin of specific PD populations. Such studies provide a step for the better understanding of the pathogenesis of idiopathic PD and synucleinopathies in general, potentially leading to the eventual development of novel neuroprotective therapeutic strategies.

## AUTHOR CONTRIBUTIONS

**Ioanna Alefanti:** Conceptualization; methodology; data curation; investigation; formal analysis; writing – original draft; resources. **Christos Koros:** Conceptualization; methodology; validation; investigation; formal analysis; writing – original draft; writing – review and editing; resources. **Viktoria Tsami:** Conceptualization; methodology; data curation; investigation; validation; writing – original draft. **Athina Maria Simitsi:** Methodology; data curation; validation; investigation; formal analysis; writing – original draft; resources. **Chrisoula Kartanou:** Conceptualization; methodology; data curation; formal analysis; investigation; resources; writing – original draft. **Nikolaos Papagiannakis:** Conceptualization; methodology; supervision; data curation; formal analysis; validation; investigation; writing – review and editing. **Maria Bozi:** Conceptualization; methodology; investigation; validation; data curation; writing – review and editing; project administration. **Roubina Antonelou:** Conceptualization; investigation; methodology; data curation; writing – original draft. **Matina Maniati:** Conceptualization; methodology; data curation; formal analysis; validation; investigation; writing – review and editing. **Ann‐Kathrin Hauser:** Conceptualization; methodology; data curation; formal analysis; validation; writing – review and editing; funding acquisition. **Stefanos Varvaressos:** Conceptualization; methodology; data curation; validation; investigation; writing – review and editing. **Anastasios Bonakis:** Writing – review and editing; resources; supervision; methodology; investigation; validation. **Konstantinos Lourentzos:** Methodology; data curation; supervision; formal analysis; validation; investigation; writing – review and editing. **Periklis Makrythanasis:** Conceptualization; methodology; data curation; supervision; validation; investigation; writing – review and editing. **Sokratis G. Papageorgiou:** Writing – review and editing; validation; supervision; data curation; investigation. **Christos Proukakis:** Conceptualization; data curation; formal analysis; validation; writing – review and editing. **Constantinos Potagas:** Methodology; supervision; validation; writing – review and editing; formal analysis. **Thomas Gasser:** Methodology; data curation; investigation; validation; writing – review and editing. **Georgios Koutsis:** Project administration; methodology; data curation; investigation; resources; writing – original draft; writing – review and editing. **Georgia Karadima:** Methodology; writing – original draft; writing – review and editing; funding acquisition; data curation; investigation; project administration. **Leonidas Stefanis:** Conceptualization; writing – original draft; writing – review and editing; methodology; investigation; project administration; resources; funding acquisition; data curation.

## CONFLICT OF INTEREST STATEMENT

The authors do not report any conflict of interest related to this article.

## Supporting information


Data S1.

## Data Availability

The data that support the findings of this study are available on request from the corresponding author. The data are not publicly available due to privacy or ethical restrictions.
