# Supplementary material for: The novel p.A30G SNCA pathogenic variant in Greek patients with familial and sporadic Parkinson's disease
Source: Eur J Neurol. 2025 Jan 29;32(2):e16562. doi: 10.1111/ene.16562 (PMC11775907; doi:10.1111/ene.16562)
Supplement: Supplementary file 1 — Data S1. [file ENE-32-e16562-s001.docx]

**Supplemental material**

**Family 1**

The pedigree of family 1 (figure2a) originated from the island of Corfu, located west of mainland Greece. The index case F1-III.3 was a patient in their 60s. The first symptoms emerged at the age of 61, with complaints of generalized bradykinesia and a sensation of internal tremulousness. Additional symptoms were reported, including depression, sleep disturbances, olfactory dysfunction and constipation that preceded the onset of motor symptoms. By 3 years of onset, the patient had mild progression of motor symptoms without lateralization, and was suffering from moderate depression and anxiety. The MMSE score was 27/30. The patient’s father (II.5) suffered from PD with a late disease onset, in his 80s.

A paternal cousin, case (F1-III.1), visited the outpatient clinic as a separate patient; he was diagnosed with PD at the age of 53. Upon testing positive for the p.A30G *SNCA* mutation, it was revealed that the two cases were related. At the age of 61 years, after 8 years of disease duration, the cousin had manifested severe motor complications. In the on state, there was mild left-predominant rigidity and bradykinesia without tremor. The MDS-UPDRS-III score was 30 in the off state and the Hoehn and Yahr stage was 2. Eventually, Duodopa pump therapy was initiated, with a good response. Regarding non-motor manifestations, the patient suffered from constipation, severe olfactory deficits (Sniffin’ Sticks test: 1/12), mild visual hallucinations and mild depression. Sleep disturbances related to RBD and daytime sleepiness were also reported. The neuropsychological examination revealed mild cognitive impairment (MoCA score: 22/30), although no cognitive problems in daily life were noted 8 years after disease onset. The patient’s father, an obligate carrier given the connection with F1-III.3, passed away in his 80s without any signs of Parkinsonism.

**Family 2**

Index case (F2-II.3, figure2b) was a patient in their 60s, diagnosed with PD at the age of 49, when resting tremor on the right arm was noticed. After 13 years of disease, the patient manifested mild symmetrical rigidity, bradykinesia and tremor, predominantly of her upper extremities, along with mild hypomimia, hypophonic speech and loss of dexterity. Motor symptoms responded well to low doses of levodopa with mild motor complications. MDS-UPDRS III score (on phase) was 24. However, severe cognitive decline was observed, even on the highest dose of Rivastigmine patch, with a MoCA score of 10/30, along with depression, anxiety and apathy, and the patient was consequently unable to perform chores at home. Further examination revealed olfactory deficits, daytime sleepiness, painful sensations and autonomic dysfunction (constipation, urinary incontinence). The pedigree of the family originated from the Greek community of Istanbul and from the Greek island, Chios. No relatives with PD-related history were reported. Both parents passed away in their 80s.

**Family 3**

Index case F3-II.2 (figure2c) was a patient in their 50s. Both sides of the family originated from distant villages of the Peloponnese region in Southern Greece. The initial presentation of the disease at the age of 50 included bradykinesia and rigidity in the lower left extremity, without tremor. Motor symptoms were initially levodopa-responsive. Gradually, dyskinesia and wearing- off with freezing and dystonia manifested. At the time of the first examination, additional bradykinesia and rigidity in the upper extremities were noted but there was still no tremor. At that time, the MDS-UPDRS-III score was 9 (on), the MMSE score was 30/30 and the MoCA score was 29/30. The patient suffered from several non-motor symptoms such as depression, anxiety, hyposmia, urinary incontinence, and constipation. Depression and hyposmia were reported to be present approximately 12 years before the initiation of the disease. Over the next 3 years, severe motor complications developed, and the patient was placed on a Duodopa pump with modest response. The mother of the patient (I.1) was also diagnosed with PD at the age of 75 but tested negative for the p.A30G mutation. At the time of the examination of the proband, the mother was in her 80s, and her disease severity made her examination in the clinic impossible. The father (I.2) of the proband died in his 80s from unknown causes and the paternal family history was free of neurological disease.

**Family 4**

Index case F4-II.1 (figure2d) had already tested positive for D409H *GBA* gene mutation and initially presented with left-dominant parkinsonian symptoms at the age of 33 years. The first clinical examination at the outpatient clinic after 11 years of disease revealed moderate rigidity and postural tremor of the left extremities with a dystonic position of the left upper limp. The patient was suffering from severe dyskinesia, alternating with periods of immobility, freezing of gait and frequent falls. Additional symptoms noted included constipation, RBD and mild cognitive impairment with deficits in executive functions and attention. Despite the placement of a Duodopa pump, the motor complications were debilitating. By the age of 46 (13 years of disease) the patient became bedridden, developed severe dementia, and passed away soon after. The father of the index case (I.2) had been diagnosed with PD but no further information was available.

**Family 5**

Index case F5-II.2 (figure2e) presented with a typical PD phenotype of 6 years duration. The pedigree of the family (figure 2a) originated from the Greek community of southern Albania (unrelated, from distant villages of the Deropoli region). The first symptom emerged at the age of 58 when left upper limb rigidity was noticed. Symptoms included asymmetrical resting tremor, rigidity and bradykinesia especially on the left, as well as postural instability with frequent falls. Initial response to levodopa treatment was good but very soon motor fluctuations developed. Non-motor symptoms were also reported, including mild cognitive impairment and rapid eye movement (REM) sleep behavior disorder (RBD). The MDS-UPDRS-III score was 40 (on) and the MMSE score was 20/30. The patient passed away at the age of 66 years old from cardiovascular disease. The father (I.1) of the proband was reported to have demonstrated PD, with resting tremor and bradykinesia. The sister (II.3) of the proband was also reported to have suffered from PD from the age of 62 and passed away at the age of 70 years, but no further information or DNA of these family members was available.

**Family 6**

In family 6 (figure2f), index case F6-II.2 was a patient in their 50s, originating from the region of Central Greece, with PD of 11 years duration. The patient developed rigidity and bradykinesia of the right extremities without tremor in their 40s. Motor symptoms were initially responsive to levodopa but, after 6 years, gradually wearing off and freezing of gait developed. Clinical examination revealed moderate to severe bilateral bradykinesia and rigidity without tremor and gait dysfunction with shuffling. The MDS-UPDRS-III score was 63 (off) and the patient was at stage 3 of the H&Y scale. Various non–motor manifestations were reported such as RBD, daytime sleepiness, urinary incontinence, excessive perspiration and fatigue. Olfaction was impaired with Sniffin’ Sticks test of 4/12. Psychiatric features, including hallucinations, depression and apathy were present. The patient had significant cognitive impairment with a MoCA score of 16/30, with predominant deficits in executive functioning, delayed recall, attention and language. There was no PD-related family history. The father (I.2) of the proband passed away in his 40s.

**Family 7**

Index case F7-II.6 (figure2g) was a patient in their 60s, originating from the Attica region, who, at the age of 59, developed rest tremor of the left upper limb and bradykinesia of the left lower limb. Over the next years, rest tremor of both upper extremities developed with good response to levodopa. Clinical examination revealed rigidity, reduced arm swing of the left arm and hypomimia. Non motor symptoms, including constipation, mild dysphagia, RBD and anxiety were also mentioned. After 6 years of disease, the MDS-UPDRS III score (on) was 16 and the patient was at stage 2 H&Y. The MoCA score was 23, with deficits in delayed recall, fluency and attention. No PD family history was reported. One sister (II.3) of the index case was mentioned to have head tremor in her 70s but she did not develop other symptoms by the time she passed away, 5 years later.

**Family 8**

Index case F8-II.3 (figure2h) was a patient in their 60s with 5 years of disease who presented with rest tremor and bradykinesia of the right lower extremity. Signs of depression and RBD were mentioned to precede the initiation of the motor symptoms by 2 years. Initially, the response to levodopa treatment was good, but after 2 years wearing-off was reported, and gradually higher dose of levodopa were required. At that point the patient suffered from motor disability and mild dyskinesias, with significant cognitive impairment, treated with high dose of rivastigmine. The MDS-UPDRS III score (on) was 34 and the MoCA score 14. Visual hallucinations and orthostatic hypotension were also noted. The family pedigree originated from Central Greece and the Epirus Region. No PD related family history was mentioned.

**Family 9**

Index case F9-III.1 (figure2i) was a patient in their 40s with a family history of PD. The father and the paternal grandfather were reported to suffer from Parkinsonism. The origin of the family pedigree was from the southern Peloponnese region. The past year the patient complained of motor difficulty in the right extremities. Clinical examination revealed bradykinesia and rigidity on the right side of the body and mild bilateral hand tremor. At that time the MMSE score was 30/30. Anxiety and mild depression had been present for four years. Levodopa treatment led to a good response.
